# Tender violaceous nodules and petechia on the legs

**DOI:** 10.1016/j.jdcr.2024.07.043

**Published:** 2024-09-20

**Authors:** Subin Lim, Imene Ben Lagha, Victoria Billero, Gabriela A. Cobos, Arash Radfar, Fei-Shiuann Clarissa Yang

**Affiliations:** a Department of Dermatology, Tufts Medical Center, Boston, Massachusetts; b Department of Pathology, Tufts Medical Center, Boston, Massachusetts

**Keywords:** connective tissue disease, panniculitis, scurvy, vitamin C deficiency

## Case

**Fig 1:**
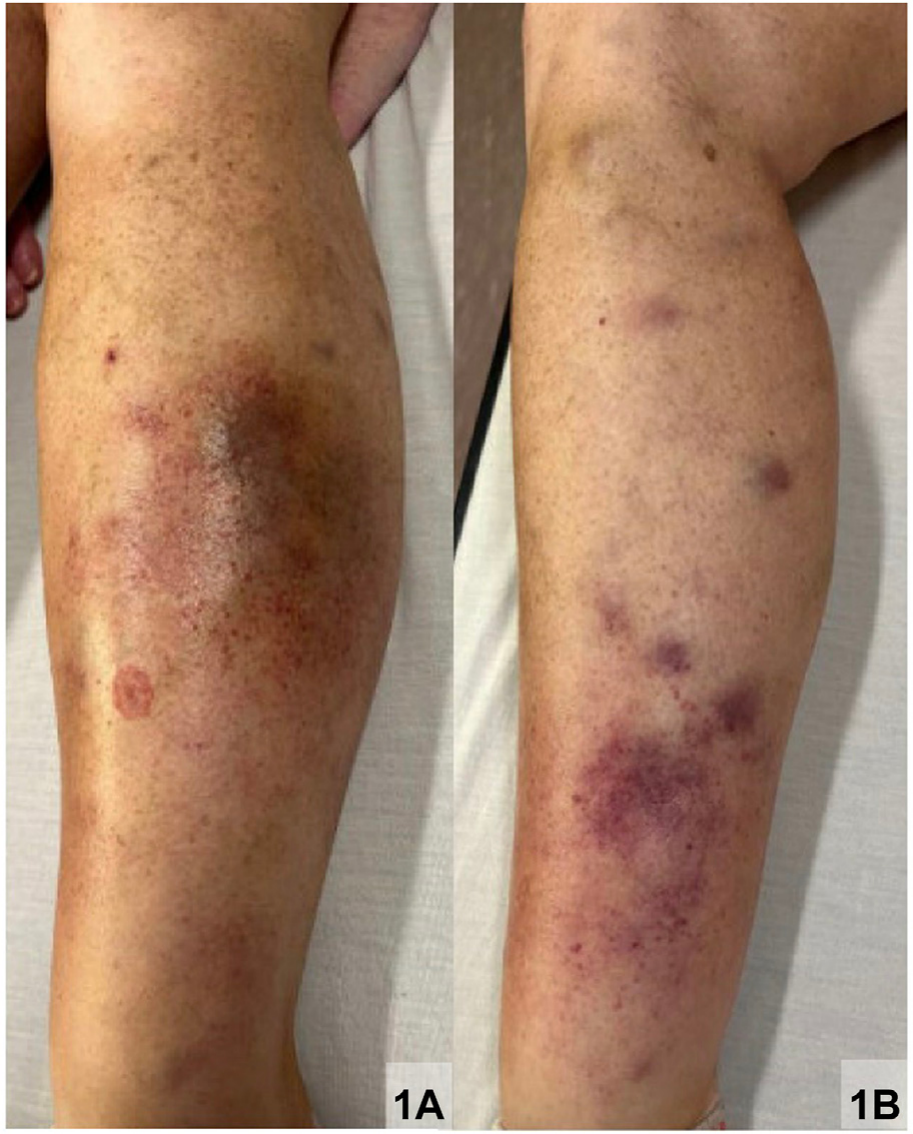
Initial clinical presentation of the patient's (**A**) left leg and (**B**) right leg.

**Fig 2:**
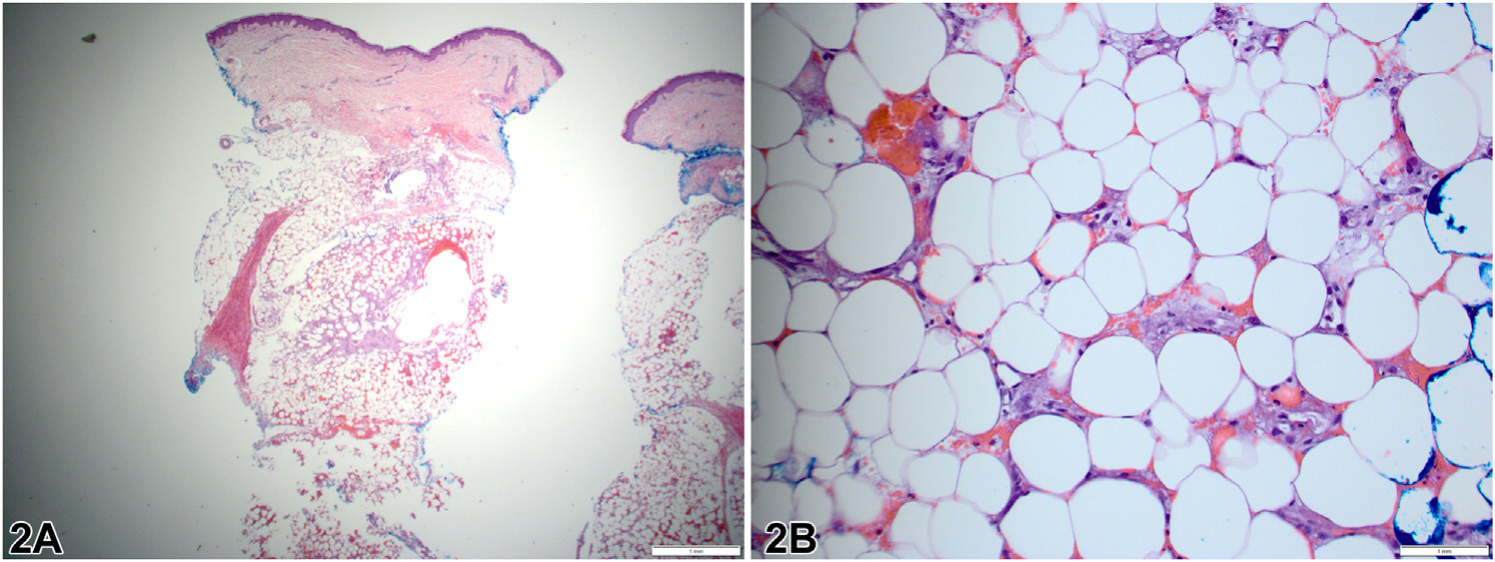
Punch biopsy findings of an indurated nodule on right medial leg, showing (**A**) changes that are limited to the subcutis, involving fat lobules and sparing the septa. **B,** prominent fat necrosis with hemorrhage, hemosiderin deposition, numerous foamy histiocytes with focal granulomas, and microcyst formation.

A 46-year-old female with hypogammaglobinemia, iron-deficiency anemia, and multiple food allergies presented with tender, indurated, violaceous nodules and petechiae predominantly on the lower extremities for 6 weeks (Fig 1). She endorsed fatigue, gingival bleeding, joint hemarthrosis, and shortness of breath. A 4-mm punch biopsy demonstrated prominent fat necrosis with hemorrhage and foamy histiocytes with focal granulomas (Fig 2). No infections were identified, and acid-fast bacilli staining was negative. Labs revealed elevated c-reactive protein, anemia, and hematuria. Platelet count, International normalized ratio, amylase, lipase, liver function tests, antineutrophil antibodies, antineutrophil cytoplasmic antibodies, and chest x-ray were unremarkable. Her medications included oral contraceptives, nonsteroidal anti-inflammatory drugs, and monthly intravenous immunoglobulin and iron infusions.

**Question 1: What is the most likely diagnosis?**

A. Erythema nodosum (EN)
B. Pancreatic panniculitis
C. Nutritional deficiency
D. Nodular vasculitis
E. Immune thrombocytopenic purpura

**Answers:**

A. Erythema nodosum (EN) – Incorrect. EN presents with tender red subcutaneous nodules on the lower extremities and may be associated with infections, oral contraceptives, and other systemic inflammatory disorders. While our patient’s nodules could represent EN, the expected histopathology would be a septal and not lobular panniculitis, as seen in our case.
B. Pancreatic panniculitis – Incorrect. Pancreatic panniculitis may present as tender nodules on the lower limbs and may be associated with arthropathy and pleural effusions. Histologically, it may also show fat necrosis and foamy histiocytes. However, contrary to typical presentations, there were no signs of abdominal pain or elevated serum amylase or lipase.
C. Nutritional deficiency – Correct. Given our patient’s history of multiple food allergies and findings of petechiae, ecchymoses, hemarthrosis, and gingival bleeding, vitamin C (ascorbic acid) levels were evaluated, revealing a striking deficiency (<0.1 mg/dl). As ascorbic acid is involved in collagen formation within the capillary walls, its deficiency can result in vessel fragility and hemorrhage, causing the skin findings seen in our patient. Consequently, vitamin C deficiency (scurvy) was diagnosed.
D. Nodular vasculitis – Incorrect. While nodular vasculitis may present with erythematous, tender nodules, they typically appear more commonly on the calves than on the shins. Histopathology often reveals lobular or septolobular panniculitis with vasculitis involving the medium-sized vessels, along with granulomatous inflammation and necrosis. Additionally, acid-fast bacilli staining was negative.
E. Immune thrombocytopenic purpura – Incorrect. Although immune thrombocytopenic purpura can present with petechiae and ecchymoses, thrombocytopenia is typically present.

**Question 2: Which of the following are risk factors for this condition?**

A. Infections and oral contraceptive use
B. Tuberculosis and autoimmune disorders
C. Pancreatitis and hypertriglyceridemia
D. Limited access to fresh foods and malabsorption disorders
E. Chronic hepatitis C and lymphoproliferative disorders

**Answers:**

A. Infections and oral contraceptive use – Incorrect. Infections (bacterial, viral, and fungal) and oral contraceptives are risk factors for EN. Hormonal changes during pregnancy can trigger EN as well.
B. Tuberculosis and autoimmune disorders – Incorrect. Tuberculosis has been historically associated with the development of nodular vasculitis. Autoimmune diseases such as systemic lupus erythematosus and rheumatoid arthritis have also been linked to nodular vasculitis.^1^
C. Pancreatitis and hypertriglyceridemia – Incorrect. Pancreatic diseases, such as pancreatitis and pancreatic cancer, can lead to pancreatic panniculitis. High levels of triglycerides in the blood are also a known risk factor for pancreatitis, indirectly increasing the risk of pancreatic panniculitis.
D. Limited access to fresh foods and malabsorption disorders – Correct. Limited access to fresh fruits and vegetables, prevalent in areas classified as food deserts, along with malabsorption conditions, are common risk factors linked to vitamin C deficiency (scurvy). Additional risk factors for scurvy include medical conditions that suppress appetite or induce nausea, alcohol and substance use disorders, eating disorders, and food allergies.^2^
E. Chronic hepatitis C and lymphoproliferative disorders – Incorrect. Chronic hepatitis C virus stands as the primary cause of mixed cryoglobulinemia. Additionally, lymphoproliferative disorders like lymphomas and multiple myeloma have commonly been associated with mixed cryoglobulinemia.^3^

**Question 3: What are the most common cutaneous manifestations of this condition?**

A. Cheilitis, glossitis, and seborrheic dermatitis
B. Follicular hyperkeratosis, petechiae, and ecchymoses
C. Generalized xerosis, follicular hyperkeratosis, and bitot spots
D. Photosensitive dermatitis and hyperpigmentation
E. Alopecia and periorificial dermatitis

**Answers:**

A. Cheilitis, glossitis, and seborrheic dermatitis – Incorrect. Cheilitis, glossitis, and seborrheic dermatitis are common cutaneous manifestations seen in vitamin B2 (riboflavin) deficiency.^4^
B. Follicular hyperkeratosis, petechiae, and ecchymoses – Correct. The most common and early cutaneous manifestations of vitamin C deficiency include follicular hyperkeratosis, scattered petechiae, and ecchymoses. Vitamin C is crucial for collagen synthesis, and deficiency can lead to blood vessel fragility and poor wound healing, thereby contributing to these skin findings.^2^^,^^4^
C. Generalized xerosis, follicular hyperkeratosis, and bitot spots – Incorrect. Although follicular hyperkeratosis may also be seen with vitamin C deficiency (scurvy), generalized xerosis and bitot spots are cutaneous manifestations mostly associated with vitamin A deficiency or phrynoderma.^4^
D. Photosensitive dermatitis and hyperpigmentation – Incorrect. While hyperpigmentation can occur in various vitamin deficiencies, dermatitis in sun-exposed areas such as the face, neck, and hands is more specific to vitamin B3 (niacin) deficiency. In particular, the Casal necklace is characterized by a circular, broad collar rash around the neck that affects dermatomes C3 and C4. If the deficiency is severe, it can manifest as Pellagra, which is characterized by the “3 D’s” – dermatitis, diarrhea, and dementia.^4^
E. Alopecia and periorificial dermatitis – Incorrect. Alopecia and periorificial dermatitis are cutaneous manifestations often seen in vitamin B7 (biotin) deficiency.^5^

## Conflicts of interest

None disclosed.
